# Online education and its effect on teachers during COVID-19—A case study from India

**DOI:** 10.1371/journal.pone.0282287

**Published:** 2023-03-02

**Authors:** Surbhi Dayal

**Affiliations:** Area of Humanities and Social Sciences, Indian Institute of Management Indore, Indore, Madhya Pradesh, India; Usak University College of Education, TURKEY

## Abstract

**Background:**

COVID pandemic resulted in an initially temporary and then long term closure of educational institutions, creating a need for adapting to online and remote learning. The transition to online education platforms presented unprecedented challenges for the teachers. The aim of this research was to investigate the effects of the transition to online education on teachers’ wellbeing in India.

**Methods:**

The research was conducted on 1812 teachers working in schools, colleges, and coaching institutions from six different Indian states. Quantitative and qualitative data was collected via online survey and telephone interviews.

**Results:**

The results show that COVID pandemic exacerbated the existing widespread inequality in access to internet connectivity, smart devices, and teacher training required for an effective transition to an online mode of education. Teachers nonetheless adapted quickly to online teaching with the help of institutional training as well as self-learning tools. However, respondents expressed dissatisfaction with the effectiveness of online teaching and assessment methods, and exhibited a strong desire to return to traditional modes of learning. 82% respondents reported physical issues like neck pain, back pain, headache, and eyestrain. Additionally, 92% respondents faced mental issues like stress, anxiety, and loneliness due to online teaching.

**Conclusion:**

As the effectiveness of online learning perforce taps on the existing infrastructure, not only has it widened the learning gap between the rich and the poor, it has also compromised the quality of education being imparted in general. Teachers faced increased physical and mental health issues due to long working hours and uncertainty associated with COVID lockdowns. There is a need to develop a sound strategy to address the gaps in access to digital learning and teachers’ training to improve both the quality of education and the mental health of teachers.

## Introduction

As of November 4, 2021, the spread of novel coronavirus had reached 219 countries and territories of the world, infecting a total of 248 million people and resulting in five million deaths [1]. In March 2020, several countries including India declared a mandatory lockdown, resulting in the temporary closure of many institutions, not least educational ones. Since then, various restrictions and strategies have been implemented to counter the spread of the virus. These include wearing masks, washing hands frequently, maintaining social and physical distance, and avoiding public gatherings. The pandemic has greatly disrupted all aspects of human life and forced new ways of functioning, notably in work and education, much of which has been restricted to the household environment. The closure for over a year of many schools and colleges across the world has shaken the foundations of the traditional structures of education. Due to widespread restrictions, employees have been forced to carve out working spaces in the family home; likewise, students and teachers have been compelled to bring classes into homes [2]. Nearly 1.6 billion learners in more than 190 countries have been physically out of school due to the pandemic. In total, 94 percent of the world’s student population has been affected by school closures, and up to 99 percent of this student population come from low-to middle-income countries [3].

According to the World Economic Forum, the pandemic has changed how people receive and impart education [4]. Physical interaction between students and teachers in traditional classrooms has been replaced by exchanges on digital learning platforms, such as online teaching and virtual education systems, characterized by an absence of face-to-face connection [5]. Online education has thus emerged as a viable option for education from preschool to university level, and governments have used tools such as radio, television, and social media to support online teaching and training [6]. Various stakeholders, including government and private institutions, have collaborated to provide teachers with resources and training to teach effectively on digital platforms. New digital learning platforms like Zoom, Google Classroom, Canvas, and Blackboard have been used extensively to create learning material and deliver online classes; they have also allowed teachers to devise training and skill development programs [7]. Many teachers and students were initially hesitant to adopt online education. However indefinite closure of institutions required educational facilities to find new methods to impart education and forced teachers to learn new digital skills. Individuals have experienced different levels of difficulty in doing this; for some, “it has resulted in tears, and for some, it is a cup of tea” [8].

Teachers have reported finding it difficult to use online teaching as a daily mode of communication, and enabling students’ cognitive activation has presented a significant challenge in the use of distance modes of teaching and learning. Teachers have also expressed concerns about administering tests with minimal student interaction [9]. Lack of availability of smart devices, combined with unreliable internet access, has led to dissatisfaction with teacher-student interaction. Under pressure to select the appropriate tools and media to reach their students, some teachers have relied on pre-recorded videos, which further discouraged interaction. In locations where most teaching is done online, teachers in tier 2 and tier 3 cities (i.e., semi-urban areas) have had to pay extra to secure access to high-speed internet, digital devices, and reliable power sources [10]. Teachers in India, in particular, have a huge gap in digital literacy caused by a lack of training and access to reliable electricity supply, and internet services. In rural or remote areas, access to smart devices, the internet, and technology is limited and inconsistent [6]. In cities, including the Indian capital Delhi, even teachers who are familiar with the required technology do not necessarily have the pedagogical skills to meet the demands of online education. The absence of training, along with local factors (for example, stakeholders’ infrastructure and socio-economic standing), contributes to difficulties in imparting digital education successfully [10]. The gap in digital education across Indian schools is striking. For example, only 32.5% of school children are in a position to pursue online classes. Only 11% of children can take online classes in private and public schools, and more than half can only view videos or other recorded content. Only 8.1% of children in government schools have access to online classes in the event of a pandemic-related restrictions [11].

The adverse effects of COVID-19 on education must therefore be investigated and understood, particularly the struggles of students and teachers to adapt to new technologies. Significant societal effects of the pandemic include not only serious disruption of education but also isolation caused by social distancing. Various studies [7, 12, 13] have suggested that online education has caused significant stress and health problems for students and teachers alike; health issues have also been exacerbated by the extensive use of digital devices. Several studies [6, 11, 14] have been conducted to understand the effects of the COVID lockdown on digital access to education, students’ physical and emotional well-being, and the effectiveness of online education. However, only a few studies [13, 15–17] have touched the issues that teachers faced due to COVID lockdown.

In this context, this study is trying to fill existing gaps and focuses on the upheavals that teachers went through to accommodate COVID restrictions and still impart education. It also provides an in-depth analysis of consequences for the quality of education imparted from the teachers’ perspective. It discusses geographical inequalities in access to the infrastructure required for successful implementation of online education. In particular, it addresses the following important questions: (1) how effectively have teachers adapted to the new virtual system? (2) How has online education affected the quality of teaching? (3) How has online education affected teachers’ overall health?

## Method

Because of lockdown restrictions, data collection for this study involved a combination of qualitative and quantitative methods in the form of online surveys and telephonic interviews. A questionnaire for teachers was developed consisting of 41 items covering a variety of subjects: teaching styles, life-work balance, and how working online influences the mental and physical well-being of teachers. In the interviews, participants were asked about their experiences of online teaching during the pandemic, particularly in relation to physical and mental health issues. A pilot study was conducted with thirty respondents, and necessary changes to the items were made before the data collection. The survey tool was created using google forms and disseminated via email, Facebook, and WhatsApp. A total of 145 telephonic interviews were also conducted to obtain in-depth information from the respondents.

The data were collected between December 2020 and June 2021. The Research Advisory Committee on Codes of Ethics for Research of Aggrawal College, Ballabhgarh, Haryana, reviewed and approved this study. A statement included in the google survey form as a means of acquiring written consent from the participants. Information was gathered from 1,812 Indian teachers in six Indian states (Assam, Haryana, Karnataka, Madhya Pradesh, New Delhi, and Rajasthan) working in universities, schools, and coaching institutions. Nearly three-quarters of the total sample population was women. All participants were between the ages of 18 and 60, with an average age of 34 and a clear majority being 35 or younger. Nearly three-quarters of participants work in private institutions (25% in semi-government entities and the remainder in government entities). In terms of education, 52% of participants have a graduate degree, 34% a postgraduate degree, and 14% a doctorate. Table 1 summarizes the demographic characteristics of the participants.

**Table 1:** Demographic details of participants.

Location/state	Assam	Haryana	Karnataka	Madhya Pradesh	New Delhi	Rajasthan	Total participants
	102 (6%)	426 (24%)	16 (1%)	202 (11%)	500 (28%)	566 (31%)	1812
Age group	< 25 years	26–35 years	35–45 years	> 45 years			
	645 (36%)	456 (25%)	465 (26%)	246 (14%)			1812
Gender	Male	Female					
	458 (26%)	1354 (74%)					1812
Type of employer	Govt.	Semi-govt.	Private				
	103 (6%)	438 (25%)	1271 (69%)				1812

## Results & discussion

Upon analyzing the survey responses, three crucial areas were identified for a better understanding of the effect of COVID-19 on the Indian education system and its teachers: how effectively teachers have adapted, how effective teaching has been, and how teachers’ health has been affected.

### 1. How effectively have teachers adapted to the new virtual system?

The first research question concerns how willing teachers were to embrace the changes brought about by the online teaching system and how quickly they were able to adapt to online modes of instruction. This information was gathered from December 2020 to June 2021, at which point teachers had been dealing with school lockdowns for months and therefore had some time to become conversant with online teaching.

While 93.82% of respondents were involved in online teaching during the pandemic, only 16% had previously taught online. These results were typically different from the results of a similar study conducted in Jordon where most of the faculty (60%) had previous experience with online teaching and 68% of faculty had also received formal training [16]. Since the spread of COVID-19 was rapid and the implementation of the lockdown was sudden, government and educational institutions were not prepared for alternative modes of learning, and teachers needed some time for adjustment. Several other factors also affected the effectiveness of the transition to online education, namely access to different types of resources and training [18].

#### a. Access to smart devices

Online teaching requires access to smart devices. A surprising number of teachers stated that they had internet access at home via laptops, smartphones, or tablets. A more pertinent question, however, was whether they had sole access to the smart device, or it was shared with family members. Only 37.25% of those surveyed had a device for their exclusive use while others shared a device with family members, due to lack of access to additional devices and affordability of new devices. During the lockdown, an increase in demand led to a scarcity of smart devices, so that even people who could afford to buy a device could not necessarily find one available for purchase. With children attending online classes, and family members working from home, households found it difficult to manage with only a few devices, and access to a personal digital device became an urgent matter for many. Respondents admitted to relying on their smartphones to teach courses since they lacked access to other devices. Teachers on independent-school rosters were significantly better equipped to access smart devices than those employed at other types of schools. The data also indicates that teachers in higher education and at coaching centers had relatively better access to laptops and desktop computers through their institutions, whereas teachers in elementary and secondary schools had to scramble for securing devices for their own use.

#### b. Internet access

Internet access is crucial for effective delivery of online education. However, our survey shows that teachers often struggled to stay connected because of substantial differences between states in the availability of internet. Of the respondents, 52% reported that their internet was stable and reliable, 32% reported it to be satisfactory and the rest reported it to be poor. Internet connectivity was better in the states of Karnataka, New Delhi, and Rajasthan than in Assam, Haryana, and Madhya Pradesh. Internet connectivity in Assam was particularly poor. Consequently, many teachers with access to advanced devices were unable to use them due to inadequate internet connection.

The following comments from a teacher in Assam capture relevant situational challenges: “I do not have an internet modem at home, and teaching over the phone is difficult. My internet connection is exhausted, and I am unable to see or hear the students.” Another teacher from Haryana reported similar difficulties: “During the lockdown, I moved to my hometown, and I do not have internet access here, so I go to a nearby village and send videos to students every three days.” Another teacher from Madhya Pradesh working at a premier institution reported experiencing somewhat different concerns: “I am teaching in one of the institute’s semi-smart classrooms, and while I have access to the internet, my students do not, making it difficult to hear what they are saying.”

These responses indicates clearly that it is not only teachers living in states where connectivity was poor who experienced difficulties in imparting education to students; even those who had good internet connectivity experiences problems caused by the poor internet connections of their students.

#### c. Tools for remote learning

Teachers made use of a variety of remote learning tools, but access to these tools varied depending on the educator’s affiliation. Teachers at premier institutions and coaching centers routinely used the Zoom and Google Meet apps to conduct synchronous lessons. Teachers at state colleges used pre-recorded videos that were freely available on YouTube. Teachers in government schools used various platforms, including WhatsApp for prepared material and YouTube for pre-recorded videos. To deliver the content, private school teachers used pre-recorded lectures and Google Meet. In addition to curriculum classes, school teachers offered life skill classes (for example, cooking, gardening, and organizing) to help students become more independent and responsible in these difficult circumstances. In addition to online instruction, 16% of teachers visited their students’ homes to distribute books and other materials. Furthermore, of this 36% visited students’ homes once a week, 29% visited twice a week, 18% once every two weeks, and the rest once a month. Additionally, a survey done on 6435 respondents across six states in India reported that 21% teachers in schools conducted home visits for teaching children [19].

#### d. Knowledge and training for the use of information and communication technologies

With the onset of the pandemic, information and communication technology (ICT) became a pivotal point for the viability of online education. The use of ICT can facilitate curriculum coverage, application of pedagogical practices and assessment, teacher’s professional development, and streamlining school organization [20]. However, the effective adoption and implementation of ICT necessitated delivery of appropriate training and prolonged practice. Also the manner in which teachers use ICT is crucial to successful implementation of online education [21]. While countries such as Germany, Japan, Turkey, the United Kingdom, and the United States recognized the importance of ICT by integrating it into their respective teacher training programmes [22], this has not been case in India. However, there are some training programmes available to teachers once they commence working. In accordance with our survey results, the vast majority of respondents (94%) lacked any ICT training or experience. In the absence of appropriate tools and support, these teachers self-experimented with online platforms, with equal chances of success and failure.

The transition from offline to online or remote learning was abrupt, and teachers had to adapt quickly to the new systems. Our data indicate that teachers in professional colleges and coaching centers received some training to help them adapt to the new online system, whereas teachers in urban areas primarily learned on their own from YouTube videos, and school teachers in rural areas received no support at all. Overall, teachers had insufficient training and support to adjust to this completely new situation. Policy research conducted on online and remote learning systems following COVID-19 has found similar results, namely that teachers implemented distance learning modalities from the start of the pandemic, often without adequate guidance, training, or resources [23]. Similar trends have been found in the Caribbean, where the unavailability of smart learning devices, lack of or poor internet access, and lack of prior training for teachers and students hampered online learning greatly. Furthermore, in many cases the curriculum was not designed for online teaching, which was a key concern for teachers [24]. Preparing online lectures as well as monitoring, supervising and providing remote support to students also led to stress and anxiety. Self-imposed perfectionism further exacerbated these issues while delivering online education [15]. A study conducted on 288 teachers from private and government schools in Delhi and National Capital Region area, also found that transition to online education has further widened the gap between pupils from government and private schools. It was more difficult to reach students from economically weaker sections of the society due to the digital divide in terms of access, usage, and skills gap. The study also found that even when teachers were digitally savvy, it did not mean that they know how to prepare for and take online classes [10].

### 2. How has online education affected the quality of teaching?

Once teachers had acquired some familiarity with the online system, new questions arose concerning how online education affected the quality of teaching in terms of learning and assessment, and how satisfied teachers were with this new mode of imparting education. To address these questions, specific questionnaire items about assessment and effectiveness of teaching has been included.

#### a. Effectiveness of online education

Respondents agreed unanimously that online education impeded student-teacher bonding. They reported several concerns, including the inattentiveness of the majority of the students in the class, the physical absence of students (who at times logged in but then went elsewhere), the inability to engage students online, and the difficulty of carrying out any productive discussion given that only a few students were participating. Another significant concern was the difficulty in administrating online tests in light of widespread cheating. In the words of one teacher: “I was teaching a new class of students with whom I had never interacted in person. It was not easy because I could not remember the names of the students or relate to them. Students were irritated when I called out their names. It had a significant impact on my feedback. I would like us to return to class so I do not have to manage four screens and can focus on my students and on solving their problems.”

For these reasons, 85.65% of respondents stated that the quality of education had been significantly compromised in the online mode. As a result, only 33% reported being interested in continuing with online teaching after COVID-19. The results show slightly higher dissatisfaction in comparison to another study conducted in India that reported 67% of teachers feeling dissatisfied with online teaching [25]. Findings of this study were similar to the findings of a survey of lecturers in Ukraine assessing the effectiveness of online education. Lower quality student work was cited as the third most mentioned problem among the problems cited by instructors in their experience with online teaching, right behind unreliable internet connectivity and the issues related with software and hardware. Primary reasons for lower quality student work were drop in the number of assignments and work quality as well as cheating. Almost half (48.7%) of the participants expressed their disapproval of online work and would not like to teach online [26].

Due to the nature of the online mode, teachers were also unable to use creative methods to teach students. Some were accustomed to using physical objects and role-playing to engage students in the classroom, but they found it extremely difficult to make learning exciting and to engage their students in virtual space. Similar trends have been reported in Australia, where schoolteachers in outback areas did not find online education helpful or practical for children, a majority of whom came from low-income families. The teachers were used to employing innovative methods to keep the students engaged in the classroom. However, in online teaching, they could not connect with their students using those methods, which significantly hampered their students’ progress. Some teachers mentioned difficulties with online teaching caused by not being able to use physical and concrete objects to improve their instructions [27].

#### b. Online evaluation

Of our respondents, 81% said that they had conducted online assessments of their students. Teachers used various online assessment methods, including proctored closed/open book exams and quizzes, assignment submissions, class exercises, and presentations. Teachers who chose not to administer online assessments graded their students’ performance based on participation in class and previous results.

Almost two-thirds of teachers who had administered online assessments were dissatisfied with the effectiveness and transparency of those assessments, given the high rates of cheating and internet connectivity issues. They also reported that family members had been helping students to cheat in exams because they wanted their children to get higher grades by any means necessary. In response, the teachers had tried to devise methods to discourage students and their families from cheating, but they still felt powerless to prevent widespread cheating.

As one respondent stated: “We are taking many precautions to stop cheating, such as asking to install a mirror behind the student and doing online proctoring, but students have their ways out for every matter. They disconnect the internet cable or turn it off and reconnect it later. When we question them, they have a connectivity reason ready”.

Teachers are also concerned about the effects of the digital skills gap on their creation of worksheets, assessments, and other teaching materials. As a result, some private companies have been putting together teacher training programs. The main challenge pertains to be implementation of a type of specialized education that many teachers are unfamiliar with and unwilling to adopt [28]. Because of the lack of effective and transparent online assessments, school teachers have reported that students were promoted to the next level regardless of their performance. Thus, only time will tell how successful online education has been in terms of its effects on the lives of learners.

### 3. How has online education affected teacher’s overall health?

The onset of the COVID-19 pandemic brought about a situation that few people had experienced or even imagined living through. Governments and individuals tried their best to adjust to the new circumstances, but sudden lockdown, confinement to the household periphery, and working from home had adverse effects on the mental and physical health of many people, including educators and students. To clarify the effects of online education on teachers’ overall health, a number of questionnaire items were focused on respondents’ feelings during the lockdown, the physical and mental health issues they experienced, and their concerns about the future given the uncertainty of the present situation.

#### a. Physical health issues

COVID-19 brought a multitude of changes to the lives of educators. Confinement to the household, working from home, and an increased burden of household and caregiving tasks due to the absence of paid domestic assistants increased physical workload and had corresponding adverse effects on the physical health of educators.

**Fig 1:**
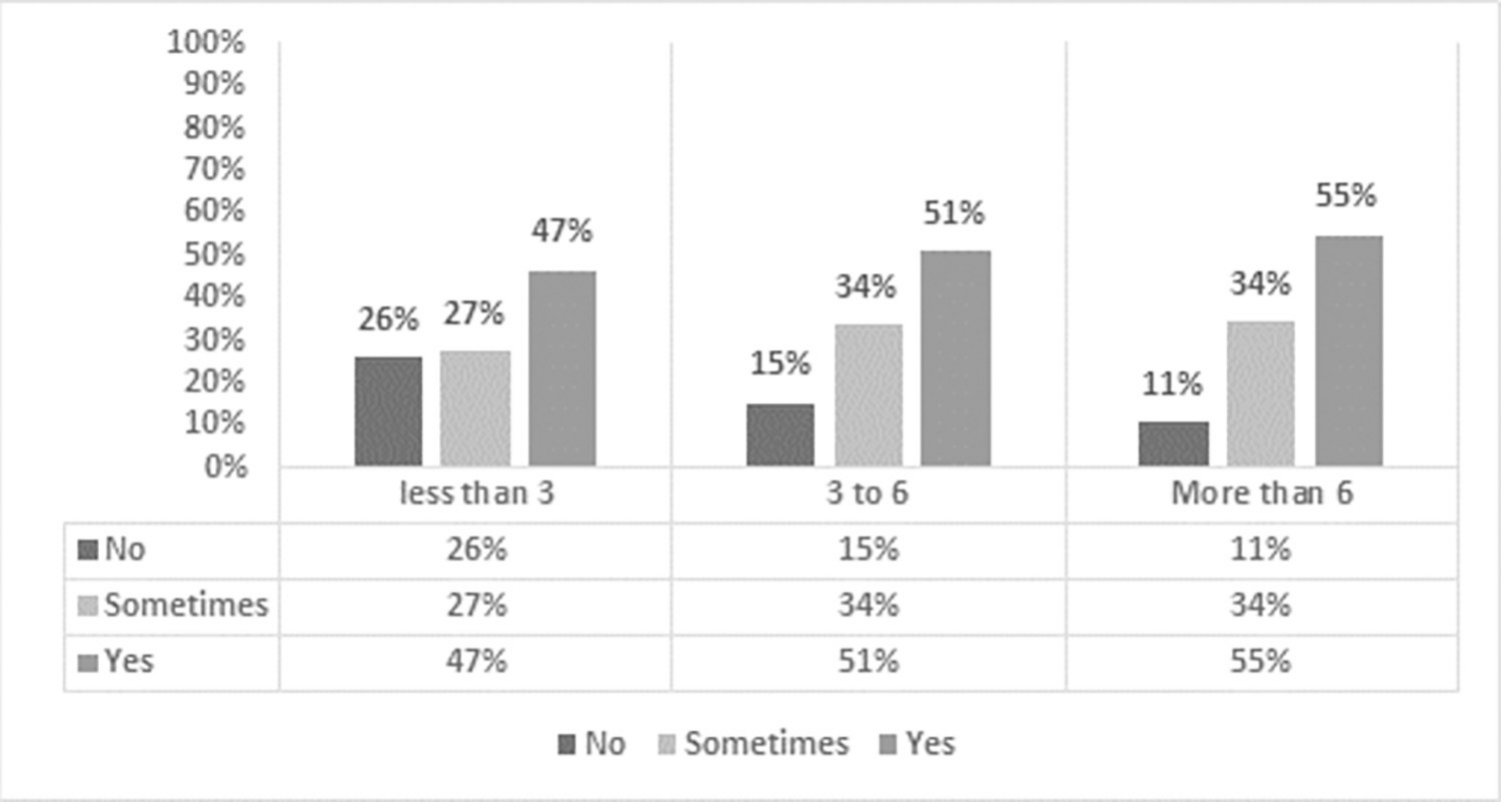
Number of working hours and frequency of physical issues.

Of the study participants, 82% reported an increase in physical health issues since the lockdown (Fig 1). Notably, 47% of those who were involved in digital mode of learning for less than 3 hours per day reported experiencing some physical discomfort daily, rising to 51% of teachers who worked online for 4–6 hours per day and 55% of teachers who worked more than 6 hours per day. Respondents reported a variety of physical health issues, including headaches, eye strain, back pain, and neck pain.

The number of hours worked showed a positive correlation with the physical discomfort or health issues experienced. A chi-square test was applied to determine the relationship between the number of online working hours and the frequency of physical issues experienced by the participants and found it to be significant at the 0.05 level (Table 2).

**Table 2:** Frequency of physical health issues experienced and number of online working hours.

Online working	Experience of physical issues				Significance
	Yes	Sometimes	No	Total	
3 hours or less	286 (308)	168 (195)	161 (113)	615	
3 to 6 hours	464 (456)	306 (288)	140 (167)	910	df = 4
More than 6 hours	157 (144)	99 (91)	31 (53)	287	*p* < 0.00001
Total	907	573	332	1812	significant at < .05

**Fig 2:**
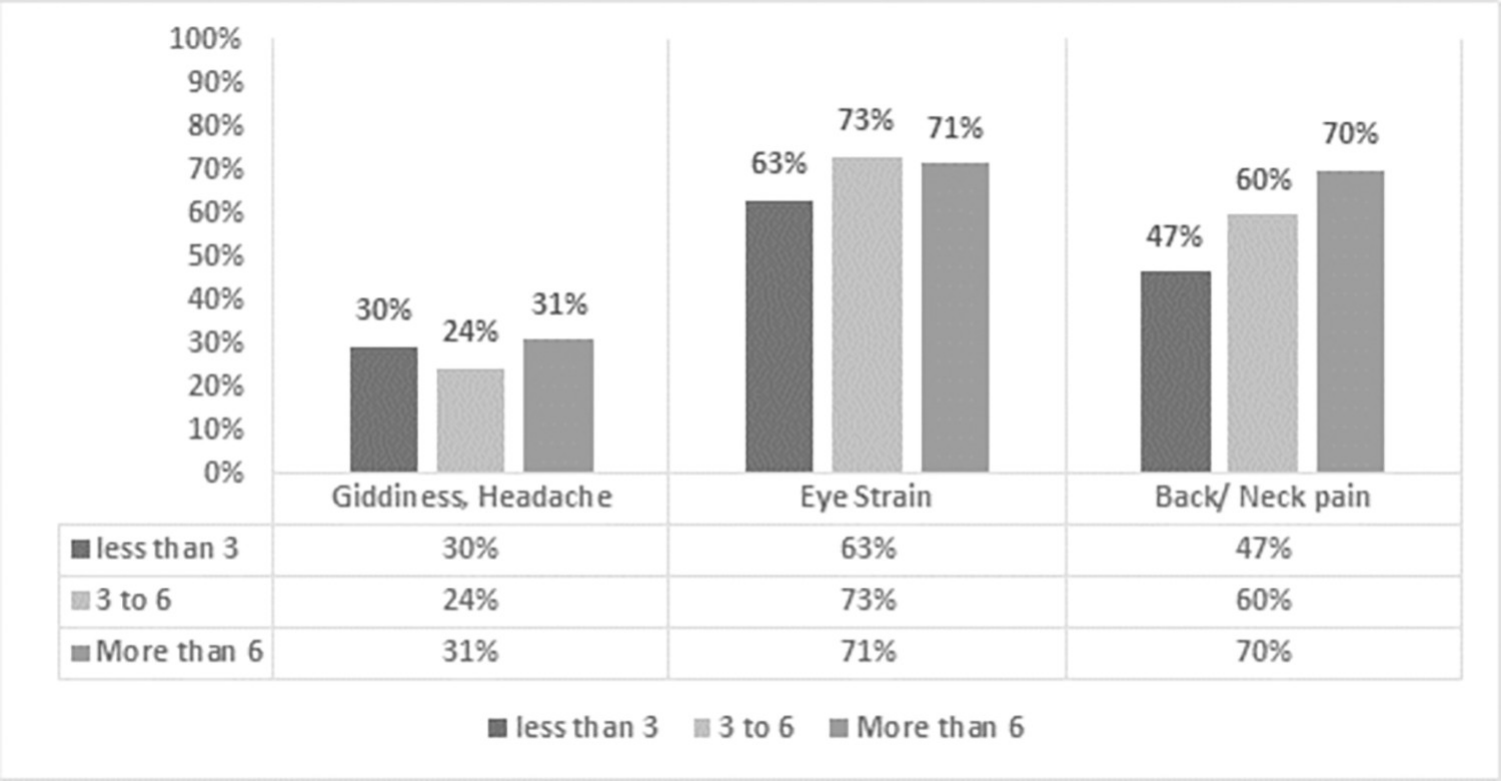
Number of working hours and types of physical health issues.

As Fig 2 shows, 28% respondents’ complaint about experiencing giddiness, headaches; 59% complain of having neck and back pain. The majority of the participants had eye-strain problems most of the time; 32% faced eye problems sometimes, and 18% reported never having any eye issue. In addition, 49% had experienced two issues at the same time and 20% reported experiencing more than 2 physical issues at the same time.

The data in this study indicates a link between bodily distresses and hours worked. As working hours increased, so did reports of back and neck pain. 47% respondents reported back and neck pain after working for 3 hours or less, 60% after working for 3–6 hours, and nearly 70% after working for 6 hours or more.

The analysis also indicates link between physical issues experienced and the educator’s gender. Women experienced more physical discomfort than men, with 51% reporting frequent discomfort, compared to only 46% of men. Only 14% of female educators reported never experiencing physical discomfort, against 30% of male educators.

In terms of types of discomfort, 76% of female teachers and 51% of male teachers reported eye strain; 62% of female teacher and 43% of male teachers reported back and neck pain; 30% of female teachers and 18% of male teachers said they had experienced dizziness and headaches. The gender differences may be caused by the increase in household and childcare responsibilities falling disproportionately on female educators compared to their male counterparts. Several studies [17, 29–31] have reported similar results, indicating that the gender gap widened during the pandemic period. The social expectations of women to take care of children increased the gender gap during the pandemic by putting greater responsibilities on women in comparison to men [29]. Women in academics were affected more in comparison to the men. Working from home burdened female educators with additional household duties and childcare responsibilities. A study done [32] in France, Germany, Italy, Norway, Sweden, the United States and the United Kingdom discovered that women were immensely affected by lockdown in comparison to men. On top of this, women with children are affected more than women without children.

No effect of age on physical discomfort was observed in this study but increasing use of online tools (such as class websites) for content creation and delivery and extended working periods were major contributors to health problems.

#### b. Mental health issues

The psychological effects of the COVID-19 pandemics have also proved difficult to manage. Being at home all day with limited social interaction, not to mention other pandemic-related sources of stress, affected the mental health of many people. The majority of the participants in this study admitted experiencing mental health issues including anxious feelings, low mood, restlessness, hopelessness, and loneliness. According to UNESCO [33], due to the sudden closure of schools and adaptability to new systems, teachers across the world are suffering from stress. Studies conducted in various parts of the world confirmed similar trends [34, 35]. In Israel, teachers reported psychological stress due to online teaching. 30.4% teachers reported being stressed in comparison to 6.1% teachers in traditional classroom settings [34]. In Spain, teachers experienced various kinds of mental health issues like anxiety, stress, and depression [36]. An Arabian study found an increased number of cases related to anxiety, depression, and violence during the pandemic [37]. In New Zealand teachers in Higher education reported being overwhelmed due to the online teaching [15].

**Fig 3:**
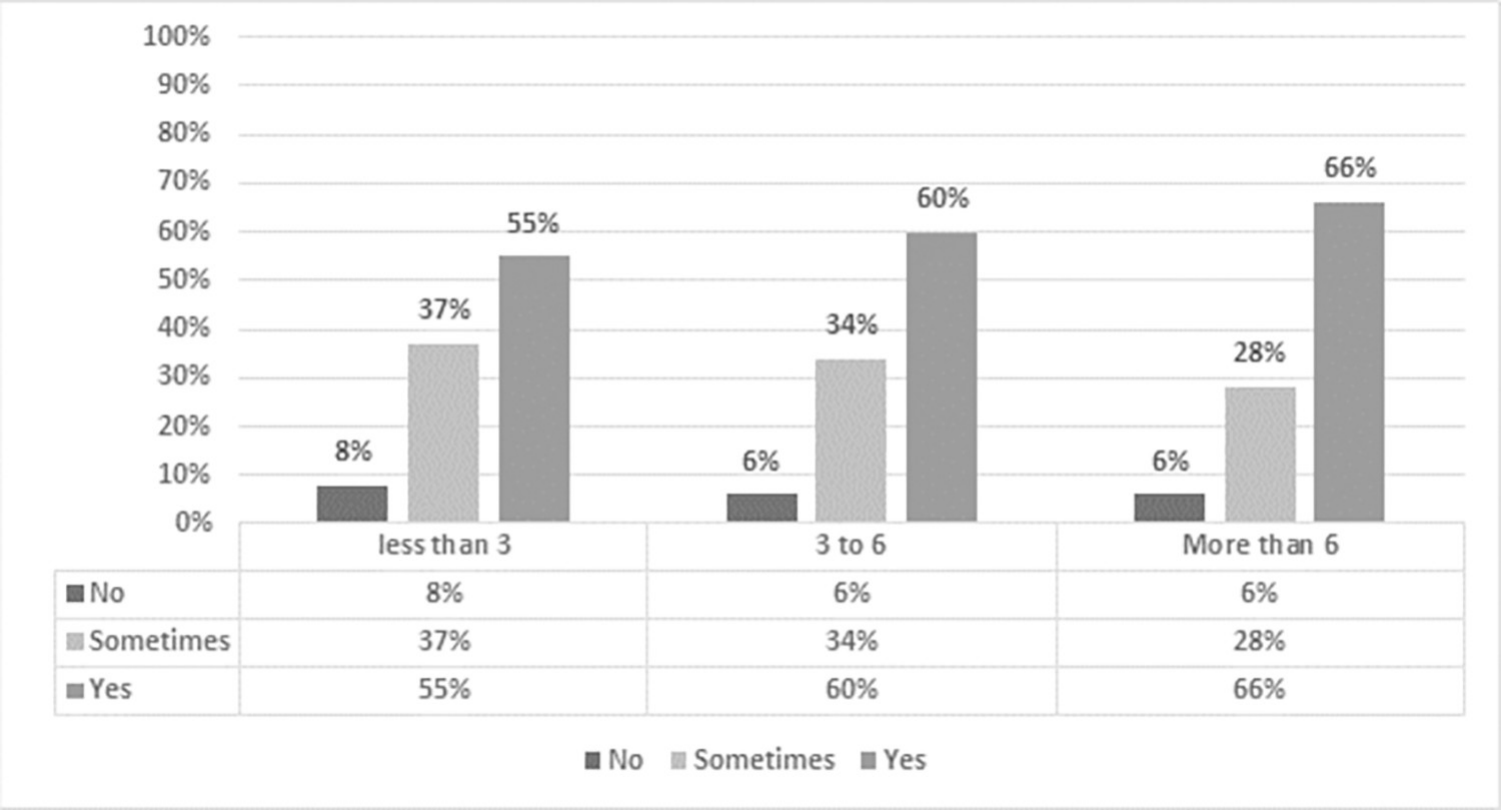
Number of online working hours and frequency of facing mental issues.

Online teaching appears to have negatively affected the mental health of all the study participants. Women (94%) reported more mental health issues than men (91%), as shown in Fig 3. Nearly two-thirds of participants said they had been dealing with mental health issues regularly and a third occasionally; only 7% said they never dealt with them. Findings of this study are in line with other studies which found that female teachers had higher levels of stress and anxiety in comparison to men [36]. Studies conducted in China reported that teachers developed mental health issues due to online classes [37, 38].

Our analysis indicated a positive relationship between the number of working hours and the frequency of mental health issues. Of the respondents who worked online for less than 3 hours, 55% experienced some kind of mental health issue; this rose to 60% of participants who worked online for 3–6 hours, and 66% of those who worked more than 6 hours every day. A chi-square test was applied to determine the relationship between the number of online working hours and the frequency of mental issues experienced by the participants and found it to be significant at the 0.05 level (Table 3).

**Table 3:** Frequency of mental health issues experienced and number of online working hours.

Online working	Experience of mental issues				Significance
	Yes	Sometimes	No	Total	
3 hours or less	339 (365)	225 (207)	51 (43)	615	
More than 3 to 6 hours	546 (540)	307 (307)	57 (63)	910	df = 4
More than 6 hours	190(170)	79 (97)	18 (20)	287	p = 0.22875
Total	1075	611	126	1812	significant at < .05

**Fig 4:**
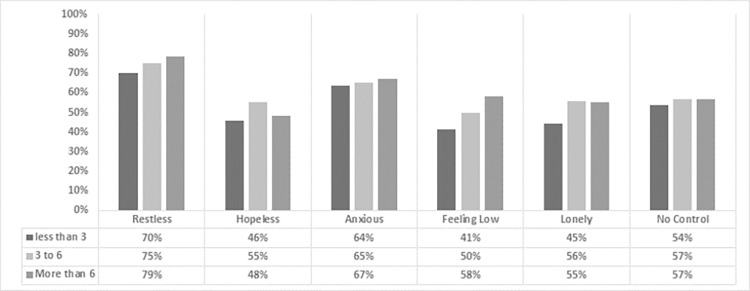
Number of working hours and types of mental health issues.

In terms of types of mental health issues, respondents reported restlessness, anxious feelings, and a sense of powerlessness, along with feelings of hopelessness, low mood, and loneliness as shown in Fig 4. The stress of adapting to a new online working environment, the extended hours of work required to prepare content in new formats, the trial-and-error nature of learning and adopting new practices, uncertainty caused by lockdown, and an overall feeling of having no control were some of the contributing factors.

Mental health issues were more common among those under the age of 35, with 64% reporting a problem most of the time compared to 53% of those over 35. It has been found that job uncertainty is one of the primary causes of a higher prevalence of mental health concerns among younger respondents than among older respondents. These findings are in line with other studies which found higher levels of stress among the young people in comparison to older one [36, 39]. Feelings of loneliness and a sense of no control were reported by 30% of respondents under the age of 35, with these feelings occurring constantly or most of the time; only 12% of respondent over the age of 35 reported experiencing these feelings always or most of the time. Of respondents under 35 years of age 61% felt lonely at some point during the COVID-19 pandemic, compared to only 40% of those age 35 or older.

This study also found gender-based differences in the frequency of mental health issues experienced, with 62% of male respondents and 52% of female respondents reporting that they had always experienced mental health issues. The types of issues also differed by gender, with men more likely to report restlessness and loneliness and women more likely to report feeling anxious or helpless. More female respondents reported feelings of hopelessness than male respondents (76% compared to 69%), and they were also more anxious (66%).

The uncertainty of the pandemic seems to have caused helplessness and anxious feelings for female teachers in particular, perhaps because a lack of paid domestic help increased the burden of household and caregiving tasks disproportionately for women at a time when the pressure to adapt to new online platforms was particularly acute. In some cases, respondents left their jobs to accommodate new family dynamics, since private employers offered no assistance or flexibility. Deterioration of mental health also led to the increased number of suicides in Japan during COVID-19 [39].

However, female teachers fared better than their male counterparts on some measures of mental health. Although half of the respondents (men and women equally) reported low mood during the pandemic, the men reported more restlessness (53%) and loneliness (59%) than the women (50% and 49%, respectively). Restrictions on eating and drinking outside the household may have had a disproportionate effect on male respondents, making them more likely to feel restless or lonely than their female counterparts, who may have handled COVID-related isolation better by being more involved in household work and caregiving.

Number of hours worked online was also a factor contributing to mental health issues. Just as respondents had more physical complaints (including eye strain, back and neck pain, and headaches) the more hours they worked online, respondents who worked longer hours online reported more mental health issues.

One of the major drawbacks of online education is the widespread occurrence of physical and mental health issues, and the results of this study corroborate concerns on this point. This study found that online teaching causes more mental and physical problems for teachers than another study, which only found that 52.7% of respondents had these problems [12].

A report by the University of Melbourne has also indicated that online teaching and learning have a negative effect on the physical and mental well-being of individuals. Teachers working from home, in particular, have reported isolation, excessive screen time, inability to cope with additional stress, and exhaustion due to increased workload; despite being wary of the risks of exposure to COVID-19, they were eager to return to the campus [27].

#### c. Support mechanisms

In general, teachers experienced good support from family and colleagues during the pandemic, with 45.64% of teachers reported receiving strong support, 29.64 percent moderate support (although the remainder claimed to have received no or only occasional support from family and colleagues). 9.39% of male respondents reported that they have never received any support in comparison to 4.36% females. Female respondents reported receiving more support than male respondents perhaps because they have access to a more extensive network of family members and coworkers. Children, parents, and siblings were cited as the provider of a robust support system by most female respondents. For example, maternal relatives called or texted children to keep them engaged and helped them with homework, and female participants said their peers helped them to prepare lectures and materials. A link was also found between age and support; the older the respondent, the stronger the support system. A possible explanation for this difference is that older people have had time to develop stronger and longer-lasting professional and personal ties than younger people.

## Conclusion

This study explored the effects of the COVID-19 pandemic on the Indian education system and teachers working across six Indian states. The effectiveness of online education methods varied significantly by geographical location and demographics based on internet connectivity, access to smart devices, and teachers’ training. While premier higher education institutions and some private institutions had provided teachers with the necessary infrastructure and training to implement effective successful online learning with relatively few challenges, teachers at schools and community colleges have more often been left to adopt a trial-and-error approach to the transition to an online system. Further, it indicates that online education has had a significant effect on the quality of education imparted and the lives and wellbeing of teachers. While online learning has enabled teachers to reach out to students and maintain some normalcy during a time of uncertainty, it has also had negative consequences. Owing to the lack of in-person interaction with and among students in digital classes, the absence of creative learning tools in the online environment, glitches and interruptions in internet services, widespread cheating in exams, and lack of access to digital devices, online learning adversely affected the quality of education. Teachers experienced mounting physical and mental health issues due to stress of adjusting to online platforms without any or minimal ICT training and longer working hours to meet the demands of shifting responsibilities. A positive correlation was found between working hours and mental and physical health problems.

The long-term impact of COVID-19 pandemic on both the education system and the teachers would become clear only with time. Meanwhile, this study sheds light on some of the issues that teachers are facing and needs to be addressed without further ado. These findings will provide direction to the policy makers to develop sound strategies to address existing gaps for the successful implementation of digital learning. However, researchers should continue to investigate the longer-term effects of COVID pandemic on online education.

## Supporting information

S1 File Supplementary material. (DOCX)
