# Supplementary material for: Online education and its effect on teachers during COVID-19—A case study from India
Source: PLoS One. 2023 Mar 2;18(3):e0282287. doi: 10.1371/journal.pone.0282287 (PMC9980775; doi:10.1371/journal.pone.0282287)
Supplement: S1 File — (DOCX) [file pone.0282287.s001.docx]

**Table 1- Involved in online teaching**

| **No** | **Yes** |
| --- | --- |
| **6.18%** | **93.82%** |

**Table 2-Involved in online teaching before COVID-19**

| **No** | **Yes** | **Grand Total** |
| --- | --- | --- |
| **84%** | **16%** | **100%** |

**Table 3-Access to digital devices**

| **Gadget** | **% of users** |
| --- | --- |
| iPad/Tab | 0.33% |
| Laptop/Desktop | 5.57% |
| Multiple | 48.51% |
| Smart Phone | 45.58% |
| **Grand Total** | **100.00%** |

**Table 4- Sharing of Gadget**

| **Gadget** | **I share it** | **Sometimes I have to share** | **I don’t share it** |
| --- | --- | --- | --- |
| iPad/Tab | 50.00% | 50.00% | 0.00% |
| Laptop/Desktop | 25.74% | 24.75% | 49.50% |
| Multiple | 27.76% | 32.08% | 40.16% |
| Smart Phone | 34.99% | 32.08% | 32.93% |
| **Grand Total** | **31.02%** | **31.73%** | **37.25%** |

**Table 5- Internet Connectivity in different regions**

| Poor | Good | Very good |
| --- | --- | --- |
| 16% | 32% | 52% |

**Table 6-Quality of internet connectivity in different regions**

| **States** | **Very Good** | **Good** | **Poor** | **Grand Total** |
| --- | --- | --- | --- | --- |
| Assam | 49.02% | 21.57% | 29.41% | 100.00% |
| Haryana | 38.26% | 42.25% | 19.48% | 100.00% |
| Karnataka | 81.25% | 12.50% | 6.25% | 100.00% |
| MP | 51.98% | 27.23% | 20.79% | 100.00% |
| New Delhi | 63.80% | 26.40% | 9.80% | 100.00% |
| Rajasthan | 52.47% | 33.39% | 14.13% | 100.00% |
| **Grand Total** | **52.26%** | **32.01%** | **15.73%** | **100.00%** |

**Table 7- Do you feel the quality of education has been compromised**

| **Yes** | **No** | **Grand Total** |
| --- | --- | --- |
| **85.65%** | **14.35%** | **100.00%** |

**Table 8- Conducting online exams**

| **Yes** | **No** | **Grand Total** |
| --- | --- | --- |
| **81%** | **19%** | **100.00%** |

**Table 9- Satisfaction with online assessment**

| **Yes** | **No** | **Grand Total** |
| --- | --- | --- |
| **29%** | **71%** | **100.00%** |

**Table 10- Will you continue online teaching after pandemic**

| **Yes** | **No** | **Grand Total** |
| --- | --- | --- |
| **33%** | **67%** | **100.00%** |

|  |  |  |  |  |
| --- | --- | --- | --- | --- |
| **Table 11-Number of working hours and type of physical health issues** | | | | |
| **No. of working hours** | Giddiness, Headache | Eye Strain | Back/ Neck pain | **Grand Total** |
| Upto 3 hrs | 30% | 63% | 47% | 615 |
| 3 to 6 hrs | 24% | 73% | 60% | 910 |
| > 6 hrs | 31% | 71% | 70% | 287 |
| Grand Total | 27% | 69% | 57% | 1812 |

**Table 12- Did you conduct online assessment**

| **Yes** | **No** | **Grand Total** |
| --- | --- | --- |
| **81%** | **19%** | **100.00%** |

**Table 13- Satisfaction with online Teaching**

| \| **Yes** \| **No** \| **Grand Total** \| \| --- \| --- \| --- \| \| **27%** \| **73%** \| **100.00%** \| | | | |  | |  |  |  |
| --- | --- | --- | --- | --- | --- | --- | --- | --- | --- | --- | --- | --- | --- | --- |
|  | | | |  | |  |  |  |
| **Table 14- Received support during Covid19** | | | | | | | |  |
| **Strong** | **Moderate** | **Sometimes** | **Never** | | **Grand Total** | | |  |
| **45.64%** | **29.64%** | **19.09%** | **5.63%** | | **100.00%** | | |  |
|  |  |  |  | |  | | |  |
| **Table15- Received support during COVID19 - based on age group** | | | | | | | | |
| **Age group** | **Strong** | **Moderate** | **Sometimes** | | **Never** | | | **Grand Total** |
| Below 25 years | 35.04% | 27.13% | 26.36% | | 11.47% | | | 100.00% |
| 26-35 years | 47.59% | 29.17% | 20.83% | | 2.41% | | | 100.00% |
| 35-45 years | 53.55% | 36.77% | 7.96% | | 1.72% | | | 100.00% |
| Above 45 years | 54.88% | 23.58% | 17.89% | | 3.66% | | | 100.00% |
| **Grand Total** | **45.64%** | **29.64%** | **19.09%** | | **5.63%** | | | **100.00%** |

**Table16- Received support during COVID19 - based on gender**

| **Age group** | **Strong** | **Moderate** | **Sometimes** | **Never** | **Grand Total** |
| --- | --- | --- | --- | --- | --- |
| Female | 47.49% | 29.32% | 18.83% | 4.36% | 100.00% |
| Male | 40.17% | 30.57% | 19.87% | 9.39% | 100.00% |
| **Grand Total** | **45.64%** | **29.64%** | **19.09%** | **5.63%** | **100.00%** |
